# An Evaluation of Two Systems for the Management of the Microbiological Quality of Water in Dental Unit Waterlines: Hygowater^®^ and IGN Calbénium^®^

**DOI:** 10.3390/ijerph18105477

**Published:** 2021-05-20

**Authors:** Damien Offner, Anne-Marie Musset

**Affiliations:** 1 INSERM (French National Institute of Health and Medical Research), UMR 1260, Regenerative Nanomedicine (RNM), FMTS, F-67000 Strasbourg, France; anne.marie.musset@chru-strasbourg.fr; 2 Faculté de Chirurgie Dentaire, Université de Strasbourg, 8 rue Ste Elisabeth, F-67000 Strasbourg, France; 3 Pôle de Médecine et Chirurgie Bucco-Dentaires, Hôpitaux Universitaires de Strasbourg (HUS), 1 Place de l’Hôpital, F-67000 Strasbourg, France

**Keywords:** disinfection, dentistry, safety, infection control, contamination, water

## Abstract

Water in dental unit waterlines (DUWL) represents a risk for vulnerable patients if its microbiological quality is not controlled. The aim of this prospective study was to evaluate two systems for its management under real conditions: Hygowater^®^ and IGN Calbenium^®^. Samples of the output water of DUWL were obtained for 5 previously contaminated units connected to Hygowater^®^, and 5 non-contaminated units connected to IGN Calbenium^®^, which was already effective for more than 1 year, as a control group. Samples were regularly collected up to 6 months after the implementation of Hygowater^®^, and were then cultured and analyzed. With IGN Calbenium^®^, except for a technical problem and a sample result in one unit at 6 months (Heterotrophic Plate Count (HPC) at 37 °C of 66 colony forming units (cfu)/mL), the results showed an absence of contamination. Hygowater^®^ took a couple of weeks to be effective on initially contaminated DUWL (over 200 cfu/mL for all the units), then showed its efficacy for 2 months (HPC at 37 °C with a mean of 40.2 ufc/mL, and HPC at 22 °C with a mean of 0.2 ufc/mL). At 6 months, results were satisfactory for HPC at 22 °C (mean of 12 ufc/mL), but HPC at 37 °C gave non-satisfactory results for 4 of the 5 units (mean of 92.2 ufc/mL). Both systems have an effect on the microbiological quality of DUWL. IGN Calbenium^®^ appears to be more reliable on a long-term basis.

## 1. Introduction

Water is an essential adjuvant in dental procedures. Indeed, it allows to cool down dental burs and scalers as well as dental tissue, and to rinse dental debris. Water flows through the dental unit waterlines (DUWL) to be brought to the site of interest, and unfortunately, microbial contamination of DUWL, via biofilms, is precisely well-described [1,2,3,4]. It is favored by the narrowness of the water tubing and the associated laminar flow at its periphery [5,6], the intermittent use of dental units [7], and the constituent material of DUWL [8]. This contamination can come either from the water supply network [9], or as a result of a back-contamination of DUWL. Indeed, many dental procedures can involve the use of water in dentistry (tooth preparation, air flow for the removal of stains and subgingival deposits, etc. [10,11]) and impact its microbiological quality in DUWL, notably linked to the backflow of oral fluids when rotary instruments stop [12,13,14]. An insufficient control of the microbiological quality of water in DUWL can jeopardize the safety of patients and of the dental team, as water is aerosolized during dental procedures [7,15], and as patients can swallow it. It is particularly important for vulnerable patients [16], as contaminated water has already led to the death of an Italian and a Swedish patient [17,18] and caused various infections in others [19]. Since water is used in almost all dental care, this represents a public health issue.

Many products regularly arrive on the market to control the microbiological quality of the water in DUWL. Many of them were evaluated, such as Alpron^®^/Bilpron^®^ with or without the association of BRS^®^ [20,21,22,23,24], Sterispray^©^ [1,4], BacTerminator^©^ [4], Oxygenal 6^©^ [1,22,23], Calbénium^©^ [1,4], Dentosept [22,23], ICX^©^ [23], or cetylpyridinium chloride [25]. Most of them are chemical treatments, used as continuous disinfection systems. In these studies, classically, the performances of the products are evaluated with the monitoring of the Heterotrophic Plate Count (HPC) at 22 and at 37 °C [4,20,22,24,25] and the search for specific pathogens such as Pseudomonas aeruginosa and the total amount of coliform bacteria [1,4,20]. Indeed, these targets are representative from both environmental and human origins, and their presence has frequently been reported in DUWL [1].

**Figure 1:**
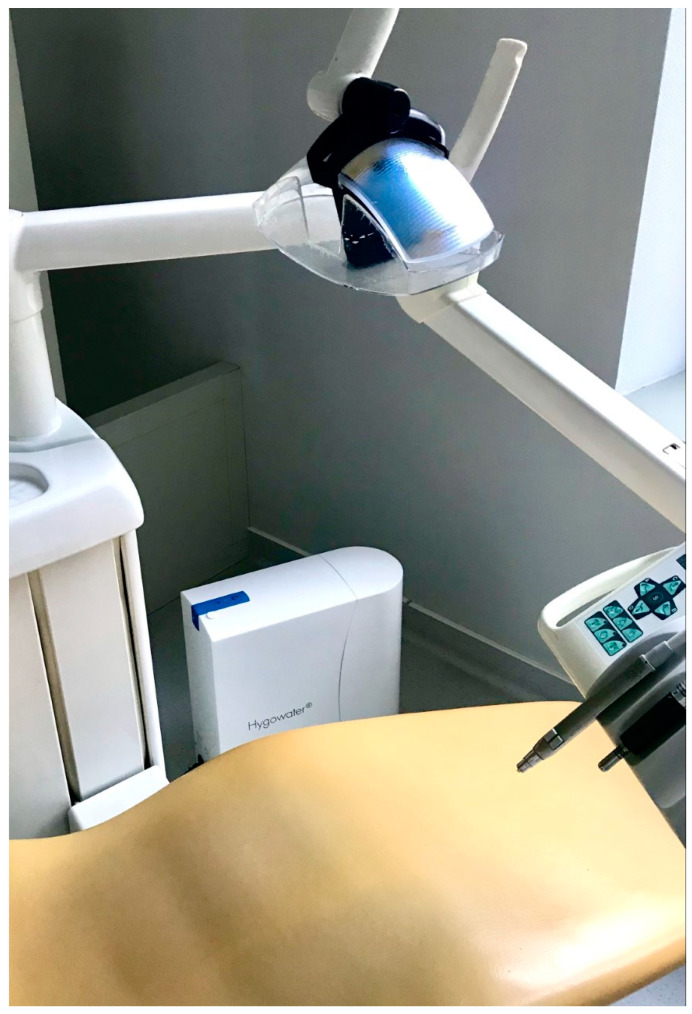
Hygowater^®^ system, connected to a dental unit.

The aim of this prospective study was to evaluate the Hygowater^®^ system (Figure 1), a physical treatment which had never been evaluated, compared with the IGN Calbenium^®^ system, regarding their performances towards the management of the microbiological quality of water in DUWL. Regarding its operating mode, which is described in the Material and Methods Section, our hypothesis was that it would be quickly effective and ensure a safe microbiological quality of the water in DUWL. The IGN Calbenium^®^ system is one of the most frequently distributed treatment systems by dental unit suppliers [1,6], has already been evaluated [1,4], and is therefore considered as a reference.

## 2. Material and Methods

### 2.1. Dental Units and Disinfection Systems

The study took place in the dental facility of the university hospital of Strasbourg, France, where patients are regularly treated by the students or the teachers. The two systems have been connected upstream from the DUWL and supplied with water of potable quality regarding the European Union’s standard [26]. This water, in a hospital environment, is permanently under microbiological monitoring to present a HPC at 22 °C below 100 colony forming units per milliliter (cfu/mL), a HPC at 37 °C below 10 cfu/mL, and an amount of coliform bacteria as well as Pseudomonas aeruginosa below 1 cfu/mL.

IGN Calbenium^®^: five units were connected to the IGN Calbenium^®^ system. One in 2019 (Calbenium unit 2), more than one year before the beginning of this study, two in 2016 (Calbenium unit 1 and 5), four years before the beginning of this study, and two in 2015 (Calbenium unit 3 and 4), five years before the beginning of this study. Thus, these dental units represented a control group. The system includes an IGN device, which allows an automatic dilution of a concentrate disinfectant liquid called Calbenium^®^ in the water supply network. It is therefore a chemical continuous disinfectant system. The precise dose of Calbenium^®^ is then automatically injected in the water flow going onto the unit shelf. The level of Calbenium^®^ is continuously monitored with an audio-visual detection system. The Calbenium^®^ solution is composed of benzalkonium chloride, EDTA, allantoin, sodium tosylchloramide, aspartame, sorbitol, and spearmint flavor. Proportions of these products are not disclosed by the manufacturer.

Hygowater^®^: five units were connected to the Hygowater^®^ system at the beginning of this study, on 1 October 2020. The system offers various filtration levels, ion-exchanges, and electrolysis processes that produce an output water, running through the DUWL, loaded with free chlorine. According to the manufacturer, the range of efficacy is between 0.2 and 1 mg/L of free chlorine. This system is therefore a physical continuous disinfectant system. The Hygowater^®^ system demands a 5 min flushing at the beginning of the day so that the water, treated and loaded with chlorine upstream of the unit, can then take place in the DUWL.

All the involved dental units underwent the same daily maintenance, matching the professional guidelines [27,28] and formalized in a specific sheet of traceability, especially for the 5 min flushing at the beginning of the day, and for the 20–30 s flushing between each patient. The units were subjected to routine activity on patients, except those for which the results of the samples were not satisfactory, for obvious safety reasons. These units were, however, regularly flushed during the day. Every Monday morning, after two days of non-utilization, the “boost” mode of the Hygowater^®^ system was activated on each unit connected with this system. It allowed a higher concentration of free chlorine to be generated for several min, and thus a shock-action. A regular flushing was then applied, during 5 min as usual at the beginning of the day.

### 2.2. Water Samples and Chlorine Follow-Up

Samples of the output water of the involved dental units were collected during the mid-day break, at different times, and after a 1 min flushing:

- D0, just before the installation of the Hygowater^®^ system. At this moment, the 5 dental units which were then connected to the Hygowater^®^ system did not benefit from any treatment. The 5 other dental units had already been connected to the IGN Calbenium^®^ system for 1 year (1 unit), 4 years (2 units), or 5 years (2 units).
- D + 1, one day after the installation of the Hygowater^®^ system.
- D + 14, only for the 5 units with the Hygowater^®^ system.
- D + 1 month, D + 2 months, and D + 6 months for every dental unit.

Table 1 describes the water samples’ culture conditions as well as the standards used [26,29,30,31]. Microbiological quality levels were set according to European directives and hospital practice [26]. They are more stringent than the standards given by the American Dental Association (ADA) [32]: satisfactory results were viable aerobic microorganism count at 22 °C ≤ 100 cfu/mL, and at 37 °C ≤ 10 cfu/mL, and the absence of specific pathogenic germs: coliform bacteria and Pseudomonas aeruginosa. In addition, to have an overview of the bacterial species incriminated in the eventual contamination of DUWL, we analyzed the nature of the culturable heterotrophic bacteria found in the samples.

Every Friday, at the mid-day break, a dosage of the free chlorine in the output water of the 5 units connected to the Hygowater^®^ system was undertaken, to ensure that the system worked correctly. The same operator proceeded each time with test strips for Hygowater^®^ products, by Dürr Dental, that were dipped for 30 s in the output water and matched to a dosimetric color chart.

Concerning the samples and threshold values, the whole present methodology joins that of previous studies [4]. As the IGN Calbenium^®^ system had precisely been evaluated in the same conditions, we have previously established that it was possible to maintain a microbiological quality of water in DUWL that is satisfactory over at least one year [4]. As such, and like in other studies, it has been used as a reference [1]. Results of the samples in units with the Hygowater^®^ system were then compared to their own anteriority and to the results of the samples in units with the IGN Calbenium^®^ system.

### 2.3. Statistical Analysis

For each time point, results of the samples in units with the Hygowater^®^ system were compared to the results at D0 and to the results of the samples in units with the IGN Calbenium^®^ system. Student’s t-tests were performed using BiostaTGV (INSERM, Paris, France, https://biostatgv.sentiweb.fr/). (accessed on 10 May 2021) *p*-values less than 0.05 were considered statistically significant.

## 3. Results

**Figure 2:**
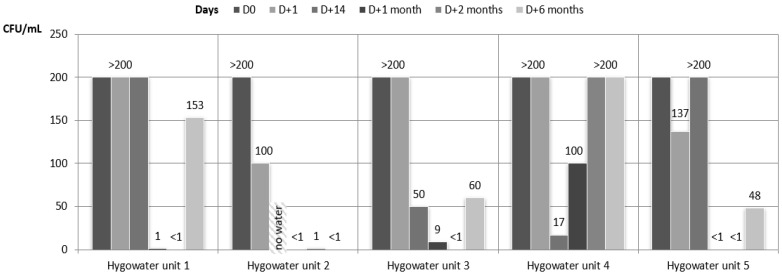
Microbiological quality of the water samples for the units connected to the Hygowater^®^ system. HPC at 37 °C.

**Figure 3:**
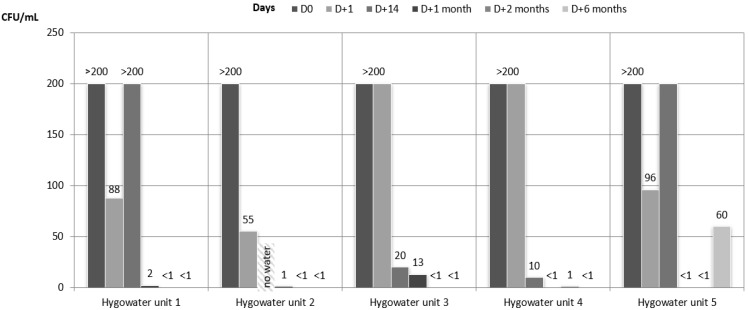
Microbiological quality of the water samples for the units connected to the Hygowater^®^ system. HPC at 22 °C.

Samples of all the dental units connected to the Hygowater^®^ system showed a high contamination before the connection to the system, at D0: HPC at 22 and at 37 °C, over 200 cfu/mL. One day after starting the Hygowater^®^ system, results still showed a high level of contamination that slightly decreased over time until D + 2 months, except for one unit (Hygowater unit 4, HPC at 37 °C). At D + 1 month and D + 2 months, all the samples showed satisfactory results, except for this same unit (Hygowater unit 4) and only for the HPC at 37 °C. Although all the results were satisfactory for the HPC at 22 °C after 6 months, the previous conform results for the HPC at 37 °C did not persist for up to 6 months. Indeed, 4 of the 5 units showed non satisfactory results (Figure 2 and Figure 3), but the decrease in the contamination versus results at D0 still was significant. The bacterial species incriminated in the contamination of DUWL are presented in Table 2. For all the samples during the whole study, the amounts of coliform bacteria and Pseudomonas aeruginosa were below 1 cfu/mL. The concentrations of the free chlorine in the output water of the 5 units connected to the Hygowater^®^ system were almost stable over the 6 months and varied between 0.4 and 1 mg/L depending on the unit and the week (Table 3). These values are proof of the correct functioning of the system, according to the recommendations of the manufacturer.

**Figure 4:**
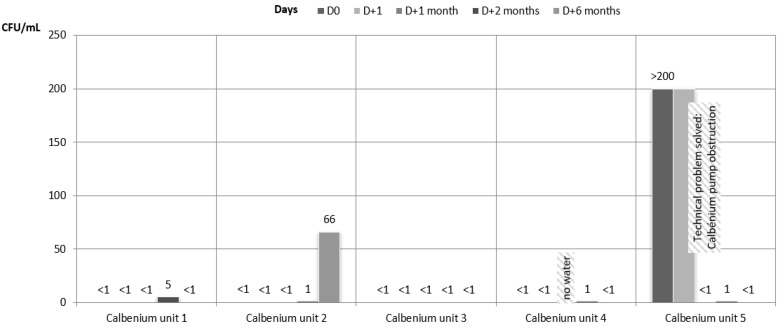
Microbiological quality of the water samples for the units connected to the IGN Calbenium^®^ system. HPC at 37 °C.

**Figure 5:**
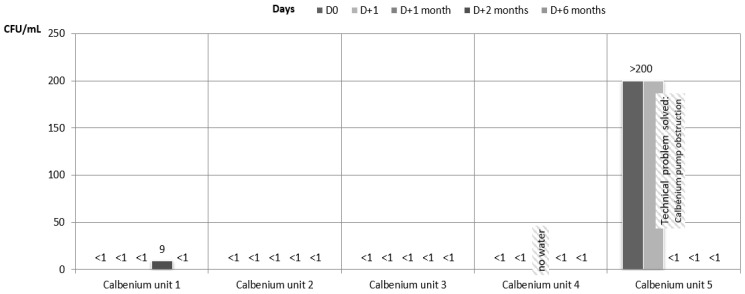
Microbiological quality of the water samples for the units connected to the IGN Calbenium^®^ system. HPC at 22 °C.

Regarding the results of the water samples from dental units connected to the IGN Calbenium^®^ system, they all showed satisfactory results during the whole study, except for one unit (Calbenium unit 2) that showed an HPC at 37 °C of 66 cfu/mL at 6 months, and for the samples at D0 and D + 1 on one other unit (Calbenium unit 5) which presented a Calbenium^®^ pump obstruction that was detected before D + 1 month. After this technical problem was solved, the results for the water samples of this specific unit (unit 5) were satisfactory (Figure 4 and Figure 5). For all the samples during the whole study, the amounts of coliform bacteria and Pseudomonas aeruginosa were below 1 cfu/mL.

At two time points, a technical problem prevented from proceeding the water sample each time on a single unit. This condition has been specified with the “no water” mention on the different figures.

Results and their statistical analysis are showed in Table 4.

## 4. Discussion

Contrary to other studies that were performed in vitro [1], the advantage of this study is that it took place under real dental care conditions. All of the units involved in this study were subjected to the same routine activity. Moreover, as its methodology joins that of a previous study, under the same conditions and with the same maintenance protocol [4], results are comparable. Still, the study presents some limitations: there are only five units involved with the Hygowater^®^ system and five with the Calbenium^®^ system, their use in real conditions represents an interesting perspective but prevents from standardized results, and finally, despite the sheet of traceability, there is no certainty that each purge lasted the recommended time. A larger number of units involved might have been interesting to get a more precise statistical analysis.

In the present study, the Hygowater^®^ system was connected to initially contaminated units, which can explain the time needed to reach satisfactory results. Indeed, active agents in water that are produced by the system had to strive with a well-implemented biofilm, whereas units equipped with the Calbenium^®^ system benefitted from the treatment for a long time: 1 to 5 years. In a previous study, however, units with contaminated water (HPC over 200 cfu/mL at 22 and 37 °C) were connected to the Calbenium^®^ system and showed satisfactory results after only one day of activity [4]. The Calbenium^®^ system is known to be an effective treatment against preformed biofilms [1] and to prevent biofilm development when used as a continuous treatment [1,4]. Our study proved this efficacy up to 5 years, except for one unit 6 months after the beginning of the study (unit 2: HPC at 37 °C of 66 cfu/mL), and sporadic technical problems. In comparison, the Hygowater^®^ system struggled to provide satisfaction up to 6 months, with results which are certainly less bad than the initial condition of the units, but do not meet the conformity criteria. Nevertheless, these results are significantly better than those for the samples at D0, and their difference with the level of contamination of units connected to the Calbenium^®^ system is not significant (Table 4). 

Another hypothesis for the time needed for the Hygowater^®^ system to produce satisfactory results is the need for 5 min of flushing every morning. Indeed, this appears to be quite a constraint, while there is little scientific support about the real efficacy of this practice [1,33], leading the Organization for Safety Asepsis and Prevention (OSAP) to recommend a 30 s flushing at the beginning of the day [33]. It is understood that the goal is not the same, since the purpose of this 5 min flushing for the Hygowater^®^ system is for water treated and loaded with chlorine to take place in the DUWL. Despite the presence of a specific sheet of traceability to follow the realization of regular flushing, which had been completed daily, there is no certainty that each purge lasted the recommended time. This is even more relevant as users were different each day because of university conditions, and as we know, the rigorous realization of flushing can be operator-dependent [6]. However, this statement is also true for the units connected to the Calbenium^®^ system. It seems that such systems which require a specific attention, like the Hygowater^®^ system, could then be more designed for a private activity with a dedicated dental staff than for large dental facilities with shared staff.

After a couple of weeks, and except for one unit that needed more than 2 months, the Hygowater^®^ system provided satisfactory results until D + 2 months, with significantly better results than at D0, and a non-significant difference with the results of units connected to the Calbenium^®^ system. Considering our results, it is not possible to assert that these results lasted until the 6th month or before. However, we can deduce that the biofilm had not been entirely neutralized, and that the efficacy of the system remains fragile. This assumption can be reinforced by the fact that the same species of bacteria are often found (Table 2), suggesting that they come from the same kind of biofilm but are expressed differently at the different time points. When compared to other studies about other water treatment systems, these results are quite equivalent, even if the methodology is not exactly comparable. The Alpron^®^ system showed compliant results (HPC at 22 °C < 100 cfu/mL) for 13 weeks in 4 of the 6 units involved in Smith’s study in 2002 [24], in 80.9% of 52 dental units after one month using the Alpron^®^ system in Chate’s study in 2010 [21], and for 98.2% of control samples that were at target level (HPC at 22 °C ≤ 100 cfu/mL, and HPC at 36 °C ≤ 10 cfu/mL) over a six-year period in the 68 units involved in Baudet’s study in 2020 [20] while associated with Bilpron^®^, using sterile water in independent tanks and with a biofilm pre-treatment using BRS^®^, which is a pre-cleaner with enzymatic agents. In a 2020 study evaluating cetylpyrimidium chloride 0.05% as a bacterial decontaminant of DUWL [25], results also showed a persistent water contamination after 7 days of treatment with a mean of 307 cfu/mL. Finally, Schel et al. led a study across the European Union in 2006 [22], in which compliant results with a threshold at HPC < 200 cfu/mL were found in 91% of 11 dental units treated with Dentosept^©^, 87% of 37 dental units treated with Alpron^®^, and 91% of 15 dental units treated with Oxygenal 6^©^. It should be noted that unfortunately, the vast majority of studies reporting water treatment system evaluations are carried out over a few weeks—a period during which the Hygowater^®^ system showed satisfactory results on 4 of the 5 units—rarely several months, and even more rarely several years [20]. Therefore, results are hardly comparable. Moreover, our study highlighted that simply treating DUWL may not be sufficient to ensure water quality, and regular monitoring must be included in the overall protocol [32].

The Calbenium^®^ system has confirmed its rapid and long-term effectiveness, here over 5 years. Still, rare contaminations and the possibility of technical problems demand this regular monitoring. Like the Hygowater^®^ system, its advantage over other previously evaluated systems is that it can be used on mains water without having to manage an external (sterile) water supply, and with an automatic functioning/dosage. Regarding the concentration of free chlorine in the water of the units connected to the Hygowater^®^ system, the amount of chlorine does not appear to be linked to the results of DUWL contamination. Indeed, for a relatively stable concentration of 0.4 mg/L during the study period (Hygowater^®^ unit 2, Table 3), the results were satisfactory, while for concentrations that regularly varied around 0.8 mg/L (Hygowater^®^ units 1 and 5, Table 3), the results indicated a stronger contamination. All these values are within the range of efficacy given by the manufacturer, and the system seems therefore not to be always sufficient against well-implemented biofilms. Thus, to be efficient even faster and over a long-term basis in initially contaminated DUWL, a complementary or a stronger treatment of biofilm before the installation of the Hygowater^®^ as a continuous treatment system should be considered and evaluated, just like the use of BRS^®^ before the continuous treatment with Alpron^®^ [20].

Another line of thought regarding the control of the microbiological quality of water in DUWL could be its biocontrol. Indeed, some microbial-based cleaning solutions showed interesting effects in the reduction of healthcare-associated infection-related pathogens [34], especially on the surfaces of a dental clinic [35]. In the same way, studies showed that predatory bacteria with protective features could be used as agents to prevent recolonization of the periodontal pocket by pathogenic germs after treatment [36]. As such, the use of probiotics could be a new potential strategy for the control of the microbiological quality in DUWL, but would demand studies to be led for its evaluation.

The results of this study can be quite generalizable, since the two systems which have been evaluated are freely marketed and used by dentists the same way they were used in this study. The units involved underwent a routine activity of dental care on patients, just like in private practices. Maintenance of units is taught and should then be equivalent in private practices, when students leave their formation. However, units’ input water used in private practices is not permanently under microbiological monitoring. Thus, results could even be worse. Finally, in real conditions, it is not possible to obtain standardized results, and this is crucial to keep in mind that a regular monitoring must be included in a maintenance protocol.

## 5. Conclusions

Both Hygowater^®^ and IGN Calbenium^®^ systems have an effect on the microbiological quality control of DUWL as continuous treatments. The IGN Calbenium^®^ system appears to be more quickly effective, and more reliable on a long-term basis. The two systems have ergonomic advantages in that they can effectively be used directly on the mains water thanks to their automatic functioning/dosage. The Hygowater^®^ system, however, presents the drawback of having to be associated with regular and rigorous flushing, more suited to a practice with a dedicated team than to a dental facility with a shared team. In initially contaminated DUWL, a complementary or stronger treatment of biofilm before the installation of the Hygowater^®^ as a continuous treatment system should be considered. 

## Figures and Tables

**Table 1:** Culture conditions of the output water samples collected from the dental units and standards used.

Microorganisms Sought	Volume Analyzed	Maximum Storage Duration Before Analysis Recommended (h)	Maximum Storage Duration Before Analysis Accepted (h)	Storage Temperature (°C) Before Analysis	Samples’ Seeding Conditions	Standards
viable aerobic MO at 22 °C	1 mL	8	12	5 ± 3	72 h at 22 °C on agar PCA by inclusion	ISO 6222 [29]
viable aerobic MO at 37 °C	1 mL	8	12	5 ± 3	48 h at 36 °C on agar PCA by inclusion	ISO 6222 [29]
Coliform bacteria and *E. coli*	100 mL	8	18	5 ± 3	24 h at 36 °C on agar TTC by membrane filtration, 2nd inspection after 48 h	ISO 9308-1 [30]
*Pseudomonas aeruginosa*	100 mL	8	12	5 ± 3 or ambient (≤25)	48 h at 36 °C on cetrimide agar by membrane filtration	ISO 16266 [31]

Abbreviations: MO, microorganism; PCA, plate count agar; TTC, tergitol = medium used for the search and count of coliform bacteria.

**Table 2:** Bacterial species incriminated in the contamination of DUWL at different time points.

	D0	D + 14	D + 1 Month	D + 2 Months	D + 6 Months
Hygowater unit 1	*Acidovorax temperans* *Sphingomonas sp.*	*Sphingomonas sp.* *Chryseobacterium sp.*	*Cupriavidus metallidurans*	-	*Blastomonas sp.* *Methylobacterium sp.*
Hygowater unit 2	*Acidovorax temperans* *Sphingomonas sp.*	-	-	-	-
Hygowater unit 3	*Acidovorax temperans* *Novosphingobium sp.* *Blastomonas sp.* *Sphingomonas sp.*	*Sphingomonas sp.*	*Blastomonas sp.* *Cupriavidus metallidurans*	-	*Methylobacterium sp.*
Hygowater unit 4	*Acidovorax temperans* *Blastomonas sp.* *Sphingomonas sp.*	*Sphingomonas sp.* *Cupriavidus metallidurans*	*Blastomonas sp.* *Methylobacterium sp.*	*Blastomonas sp.*	*Sphingomonas sp.* *Methylobacterium sp.*
Hygowater unit 5	*Blastomonas sp.* *Sphingomonas sp.*	*Blastomonas sp.* *Sphingomonas sp.* *Chryseobacterium sp.*	-	-	*Chryseobacterium sp.* *Sphingomonas sp.* *Cupriavidus sp.*
Calbenium unit 2	-	-	-	-	*Methylobacterium sp.*
Calbenium unit 5	*Stenotrophomonas maltophilia*	-	-	-	-

**Table 3:** Chlorine concentration follow-up, in mg/L (ND: No Dosage).

Weeks	Hygowater Unit 1	Hygowater Unit 2	Hygowater Unit 3	Hygowater Unit 4	Hygowater Unit 5
0	0.8	0.8	0.8	0.8	0.8
1	0.3	no water	0.4	0.3	0.6
2	0.8	0.4	0.6	0.4	0.8
3	0.8	0.4	1	0.6	0.8
4	0.8	0.4	0.8	0.8	0.6
5	0.6	0.4	0.6	0.4	0.8
6	0.8	0.4	no water	0.4	0.6
7	0.8	0.4	0.8	0.4	0.6
8	1	0.4	0.6	0.4	0.8
9	0.8	0.4	0.6	0.4	0.6
10	0.8	0.4	0.6	0.4	0.6
11	0.8	0.4	0.4	0.4	0.6
12	ND	ND	ND	ND	ND
13	ND	ND	ND	ND	ND
14	1	0.6	0.8	0.6	0.4
15	0.8	0.4	0.6	0.4	0.6
16	1	0.4	0.4	0.6	0.6
17	1	0.4	0.4	0.4	0.6
18	0.8	0.4	0.4	0.4	0.8
19	1	0.6	0.4	0.8	0.8
20	0.8	0.4	0.8	0.4	0.8
21	0.8	0.6	0.4	0.4	0.8
22	0.8	0.4	0.8	0.6	0.8
23	0.4	0.6	0.4	0.6	1
24	0.6	0.6	0.4	0.8	0.8
25	0.6	0.4	0.4	0.8	0.8

**Table 4:** Sample results (in cfu/mL) and statistical analysis.

		Unit 1	Unit 2	Unit 3	Unit 4	Unit 5	Mean Value	*p*-Value (Versus D0 in Hygowater System)	*p*-Value (Versus IGN Calbenium System)
HPC 22 °C	D0	>200	>200	>200	>200	>200	>200		***
	D + 1	88	55	200	200	96	127.8	NS	*
	D + 14	200	N/A	20	10	200	107.5	NS	N/A
	D + 1 month	2	1	13	<1	<1	3.2	***	NS
	D + 2 months	<1	<1	<1	1	<1	0.2	***	NS
	D + 6 months	<1	<1	<1	<1	60	12	***	NS
HPC 37 °C	D0	>200	>200	>200	>200	>200	>200		***
	D + 1	200	100	200	200	137	167.4	NS	**
	D + 14	200	N/A	50	17	200	116.7	NS	N/A
	D + 1 month	1	<1	9	100	<1	22	***	NS
	D + 2 months	<1	1	<1	200	<1	40.2	*	NS
	D + 6 months	153	<1	60	200	48	92.2	*	NS

(N/A: not applicable; NS: non-significant; * *p* < 0.05, ** *p* < 0.01, *** *p* < 0.001).

## Data Availability

The data presented in this study are available on request from the corresponding author.
